# The global integrative network: integration of signaling and metabolic pathways

**DOI:** 10.1007/s42994-022-00078-1

**Published:** 2022-09-21

**Authors:** Yuying Lin, Shen Yan, Xiao Chang, Xiaoquan Qi, Xu Chi

**Affiliations:** 1 Department of Dermatology, Xuan Wu Hospital, Beijing, 100053 China; 2 Agricultural Information Institute, Chinese Academy of Agricultural Science, Beijing, 100081 China; 3 Key Laboratory of Plant Molecular Physiology, Institute of Botany, Chinese Academy of Sciences, Beijing, 100093 China; 4 The Innovative Academy of Seed Design, Chinese Academy of Sciences, Beijing, 100101 China; 5 CAS Key Laboratory of Genomic and Precision Medicine, Beijing Institute of Genomics, Chinese Academy of Sciences and China National Center for Bioinformation, Beijing, 100101 China

**Keywords:** Signaling pathways, Metabolic pathways, Integration, Crosstalk of pathways

## Abstract

The crosstalk between signaling and metabolic pathways has been known to play key roles in human diseases and plant biological processes. The integration of signaling and metabolic pathways can provide an essential reference framework for crosstalk analysis. However, current databases use distinct structures to present signaling and metabolic pathways, which leads to the chaos in the integrated networks. Moreover, for the metabolic pathways, the metabolic enzymes and the reactions are disconnected by the current widely accepted layout of edges and nodes, which hinders the topological analysis of the integrated networks. Here, we propose a novel “meta-pathway” structure, which uses the uniformed structure to display the signaling and metabolic pathways, and resolves the difficulty in linking the metabolic enzymes to the reactions topologically. We compiled a comprehensive collection of global integrative networks (GINs) by merging the meta-pathways of 7077 species. We demonstrated the assembly of the signaling and metabolic pathways using the GINs of four species—human, mouse, *Arabidopsis*, and rice. Almost all of the nodes were assembled into one major network for each of the four species, which provided opportunities for robust crosstalk and topological analysis, and knowledge graph construction.

**Supplementary Information:**

The online version contains supplementary material available at 10.1007/s42994-022-00078-1.

## Introduction

Proteins are a special group of compounds in living cells, featured by their distinctive functions compared to the other compounds. The proteins involved in the biosynthesis or degradation of other compounds form the metabolic pathways, while a large number of protein enzymes sense and transfer the intra-/extra-cellular signals by signaling cascades, forming the signaling pathways. The crosstalk of signaling pathways and metabolic pathways attracts growing attentions for researches in both human disease and plants. A recent review summarized the crosstalk between signaling pathways and metabolic reprogramming in colorectal cancer facilitated by protein kinases such as AKT and c-MYC (Hon et al. 2021). Another review provided insights into the crosstalk in B cells (Jellusova 2018). The piling evidences of the crosstalk were also reviewed in the vascular biology (Uebelhoer and Iruela-Arispe 2016). For plants, the responses to the environmental stress are mostly passive, involving the production of various secondary metabolites and the downstream signaling induced by wound (Jacobo-Velázquez et al. 2015), salinity(Hartmann et al. 2015; Singhal et al. 2021), draught(Woldesemayat and Ntwasa 2018), symbiosis (MacLean et al. 2017), etc. These call for a global integrative map of prior knowledges of signaling and metabolic pathways as a reference network to elucidate the coordinated changes of cellular processes and enable the topological analysis of the crosstalk between signaling pathways and metabolic processes.

However, current pathway databases describe the signaling pathways and metabolic processes in obviously different styles. In the graph of signaling pathways, which usually refer to the cascade of post-translational modification (PTM), an edge usually connects an upstream protein (functioning as an enzyme in the reaction) and a downstream protein (representing the substrate and product of the reaction). On the other hand, an edge in a graph of metabolic pathway usually connects a substrate and a product, with the enzyme(s) sitting on the edge of the arrow. Although there are number of databases that contains both of the two types of pathways [such as KEGG (Kanehisa 2019; Kanehisa et al. 2021; Kanehisa and Goto 2000), WikiPathways (Martens et al. 2021), etc.], the displaying style of the two types of pathways still differs significantly in these databases.

The differences in the displaying style of the two types of pathways cause a series of inconveniences. First of all, they confuse the topological analysis since the edges in the two types of pathways have different meanings. Topological analysis can provide in-depth biological insight, and has been widely applied in the analysis of biological networks, i.e., the STRING database which is a popular protein–protein interaction (PPI) network database (Szklarczyk et al. 2021) and the gene co-expression network based on correlation analysis (Montenegro 2022). Second, the signaling pathways and metabolic processes are unable to be assembled into a connected graph even though there are crosstalk between them, since the enzymes of metabolic processes do not have edges which connect them to the reactions they catalyze. Of note, this also hinders the topological analysis of the integrated map of signaling and metabolic pathways. For example, KEGG displays the enzymes of metabolic reactions by putting the enzyme nodes adjacent to the edges of the reactions, while WikiPathways database provides an “anchor” attribute for each reaction in their gpml files, and the enzymes can point to these anchors, although these anchors do not have edges to the substrates or the products of the reactions.

Efforts have been made to integrate the signaling pathways and metabolic pathways. Sompairac et al. (2019) combines human signaling pathways and metabolic processes by directly merging the ACSN and ReconMap2.0 databases (Kuperstein et al. 2015; Noronha et al. 2017; Sompairac et al. 2019). However, this integration mainly focused on resolving the difficulties in visualization. The enzymes of metabolic reactions were still disconnected from the reactions they catalyze. Bag et al. (2019) integrated the signaling and metabolic pathways for epidermal growth factor receptor (EGFR)-driven glioblastoma multiforme (GBM) (Bag et al. 2019). They borrowed information from PPI network and connected the signaling proteins with the metabolic enzymes in 14 signaling pathways and 81 metabolic pathways. However, although the PPI relations connected the proteins, the proteins of the metabolic enzymes were still disconnected from their reactions. Moreover, the scope of the pathways they integrated was limited, since they only covered 27.4% of the KEGG pathways (there are currently 347 pathways available for the human species through KEGGREST). Taken together, previous efforts on the integration of signaling and metabolic pathways were limited on single species and did not fundamentally standardize the data structure, leaving the signaling and metabolic pathways topologically disconnected.

Here, we propose a novel concept, the “meta-pathway”, which standardized the basic structure of the signaling and metabolic reactions, enabling the integration of the signaling and metabolic pathway collections based on uniformed graph structure. Our newly constructed global integrative map (GIN) will open the opportunities for topological analysis of the crosstalk between signaling and metabolic networks, enabling the interpretation of multi-omics data and mining of the systemic changes at a global scope.

## Results

### Meta-pathway’s basic structure

**Fig. 1:**
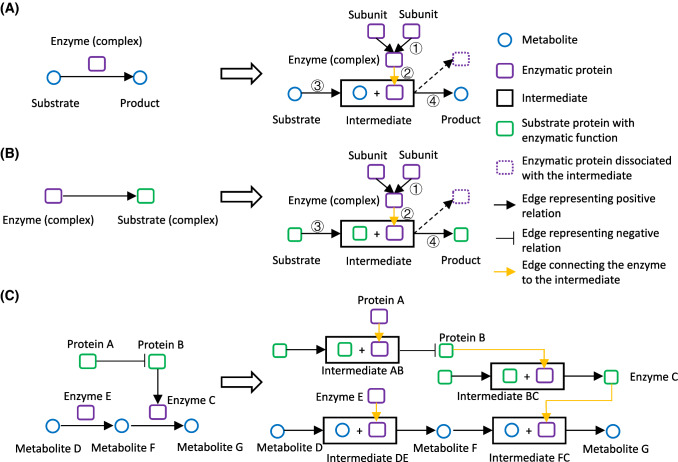
The design of meta-pathway. **A** The structure of the metabolic reactions before (left) and after (right) the conversion. If the enzyme is composed of several subunits, the relations of the subunits constituting the enzyme complex were explicitly recorded as type 1 relations. For each reaction, we introduced extra nodes representing the intermediates, which are composed of the substrate and the enzyme or enzyme complex. The type 2 relations are therefore defined as the relations of the enzyme or enzyme complex constituting the intermediate, and the similar definition goes for the type 3 relations of substrate and intermediate. The type 4 relations are defined as the conversion of the intermediate to product. The dashed arrow and box indicate the enzyme leaving the intermediate and become available for the next reaction, which we ignored to simplify the data structure. **B** The structure of the signaling pathways before (left) and after (right) the conversion. The data structure is similar to **A** after the conversion. **C** The structure of a meta-pathway with crosstalk between a signaling cascade and metabolic reactions. Before conversion (left), the interaction between the protein B and enzyme C is displayed by an edge linking the two nodes, which, however, does not link B to the metabolites, and the metabolic reactions and the signaling cascade is topologically disconnected. In the meta-pathway’s structure, the orange edge which links enzyme C to intermediate FC connects the signaling cascade and the metabolic reactions. All of the circles and boxes that appear on the actual displaying layout were annotated with plain texts

To integrate the signaling and metabolic pathways conceptually, we decomposed the common data structure of the two types of pathways into the structure of chemical reactions (Fig. 1A, B), which is universal among different biological processes. For a single metabolic reaction, we split the reaction into two steps: the formation of the intermediate and the production of the products from the intermediate (Fig. 1A, right). There are multiple advantages brought by the introduction of the intermediate. First, the intermediate is a unique identifier composed of substrate(s) with/without the enzyme(s). This resolve the chaos when there are multiple isoenzymes catalyzing the same reactions. Second, the intermediate provides a landing point of the edges starting from the enzyme(s), which is also similar to the structure of the signaling pathways (Fig. 1B, left). This helps to unify the relations of the enzymes to either metabolic substrates or signaling proteins. For a reaction in the signaling pathways, we split the reaction in a similar way (Fig. 1B, right). Of note, different from a metabolic reaction, the substrate and the product of a reaction in signaling pathway usually appear to be the same, since the major change of the substrate after the reaction is the addition/reduction of a small chemical group. Since the enzyme by definition, does not change after the reaction finishes, we did not keep the edges from intermediate to enzyme (the dashed arrows and boxes). The final form of the data structure of the meta-pathway consists of three columns, the starting node and ending node of an edge, plus a column of the type of the edges.

There are four major types of the edges, including type1, connecting the protein subunits to the protein complex, type2, connecting the enzyme to the intermediate, type3, connecting the substrate to the intermediate, and type4, connecting the intermediate to the product. Although we retained these information in the data, the final graphs constructed from the data do not discriminate the four types of the edges, since all of them represent the process of transformation of the starting nodes to the ending nodes.

Based on the design of the structure of individual signaling/metabolic reaction, we were able to build the meta-pathways integrating the heterozygous relations between molecules from signaling and metabolic pathways (Fig. 1C). For a pathway involving both signaling cascades and metabolic reactions (Fig. 1C, left), the common displaying style does not connect the reaction of B on C to the reaction of F to G topologically. Introducing the intermediate node in the graph clearly solves this problem (Fig. 1C, right), with the orange arrow (edge) connecting C to the intermediate FC.

### Construction of the global integrative networks

**Fig. 2:**
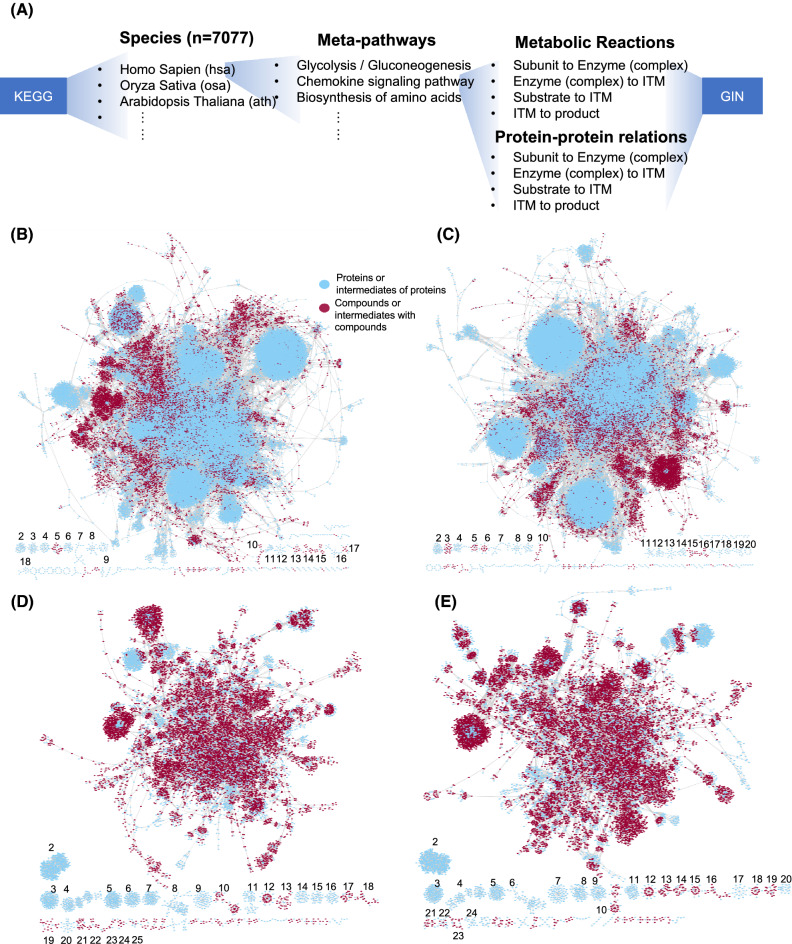
Construction of the global integrative networks. **A** The workflow of constructing GINs. The kgml files of 7077 species containing the relations of pathways were downloaded from KEGG. For each pathway of each species, we extracted the information of metabolic reactions and signaling cascades and converted them into meta-pathways. Each meta-pathway has the uniformed data structure for both of metabolic reactions and signaling cascades, then we merged the meta-pathways into the global integrative networks (GINs). ITM, intermediate. **B**–**D** The GINs of four species, human, mouse, rice and *Arabidopsis*. The nodes of compounds or intermediates containing compounds are in red. The nodes of proteins or intermediates containing no compounds are in light blue

The structure of meta-pathway makes it highly flexible, since the relations of the molecules in the pathway can be translated into the relations between nodes. Merging of the meta-pathways can be easily done by merging the nodes’ relations. We hereby built a pipeline to extract the pathways of 7077 species from KEGG and transformed them into meta-pathways, then merged the meta-pathways into a global integrative map for each species (Fig. 2A). In general, the 7077 GINs contain 45,979,621 nodes (of which are the 32,836,377 intermediates we introduced), and 110,160,537 edges (with the annotations of activation/inhibition). For illustration purpose, we selected four species: human (*Homo sapiens*), mouse (*Mus musculus*), rice (*Oryza sativa*), and *Arabidopsis* (*Arabidopsis thaliana*) for further analysis. Most of the pathways of each species can be assembled into a major network, respectively (Fig. 2B–E). To depict the distribution of the metabolites, we painted the nodes that are or include compounds in red. It is obvious that the current knowledge of the metabolic pathways in plants such as rice and *Arabidopsis* collected by KEGG is relatively more abundant than in human and mouse. However, the assembly of human and mouse are more complete than rice and *Arabidopsis*, since several signaling and metabolic pathways with large number of nodes are not connected to the major network in the GIN of rice and *Arabidopsis*. This is partially due to the fact that there are only two signaling pathways for *Arabidopsis* and rice, but more than 50 for human and mouse. Nevertheless, our work of the GIN of the four species clearly show the integration of the signaling and metabolic pathways, which provide ample resources for topological network analysis, crosstalk analysis and knowledge graph construction.

### Assessment of the network assembly

For the GINs of the two species of mammals, the proportion of the compounds and compounds related intermediates is only approximately 20%, suggesting a large proportion of the major networks are composed of the complex signaling networks in mammals. However, in rice and *Arabidopsis*, the proportion of the compounds and compounds related intermediates is > 70%, indicating that most of the known pathways in these two species are related to metabolic processes.

**Fig. 3:**
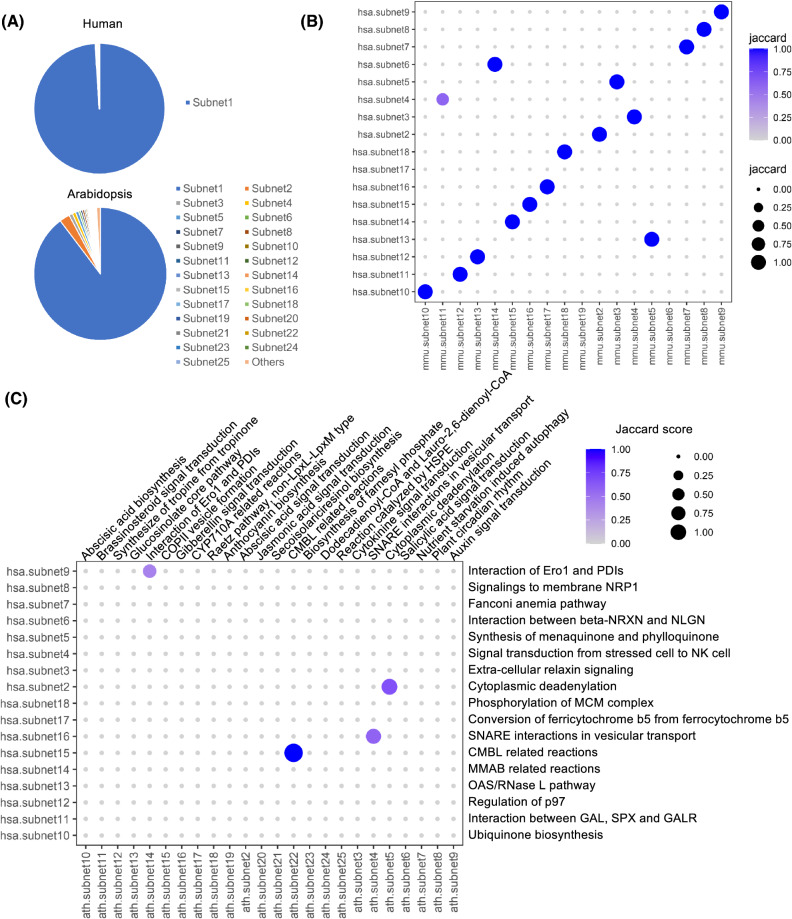
Assessment of the assembly of GINs. **A** The proportion of the subnets in human (upper) and *Arabidopsis* (lower). For the pie-chart of human’s GIN, the subnets were too small as they did not show on the chart other than subnet 1, therefore we only displayed the legend for subnet 1. **B** The Jaccard score matrix calculated using human’s and mouse’s subnets, excluding subnet 1. **C** The Jaccard score matrix calculated using human’s and Arabidopsis’s subnets, excluding subnet 1. The annotations of each subnet were manually curated

One of our major purposes to construct global integrative networks is to build a topological graph to connect as many biological processes as possible. However, the assemblies of rice’s and Arabidopsis’ GINs left out several large pieces of sub-networks. To assess the completeness of the GINs, we labeled the sub-networks (*n* > 10) numerically, with the largest sub-network designated to be subnet 1 (Fig. 2B–E). The subnet 1 of human and mouse included more than 99% of the total number of nodes, while this number dropped to 89% in *Arabidopsis* and 91% in rice (Fig. 3A and Online Resource 1, Fig. S1 a, b). To assess the homology of these subnet pieces, we converted the genes within these subnets into the KEGG Orthology (KO) ids, and calculated the Jaccard Index between the subnet considering the KO ids (for genes) and the compounds (for metabolites). The results showed that the subnet pieces of human and mouse are almost the same, with only three subnets (human subnet 17, mouse subnet 6 and 19) did not have matched subnets (Fig. 3B). Similar results were obtained for rice and *Arabidopsis* (Online Resource 1, Fig. S2). However, the homology between the mammals and plants was very low, with only four subnets matched between human and *Arabidopsis* (Fig. 3C). Further manual annotations revealed that these four subnets were interaction of endoplasmic reticulum oxidoreductin 1 (Ero1) and protein disulfide isomerases (PDIs), cytoplasmic deadenylation, soluble N-ethylmaleimide-sensitive factor attachment protein receptor (SNARE) interactions in vesicular transport, and carboxymethylenebutenolidase (CMBL) related reactions.

### Crosstalk analysis of TCA cycling pathway and MAPK signaling pathway

**Fig. 4:**
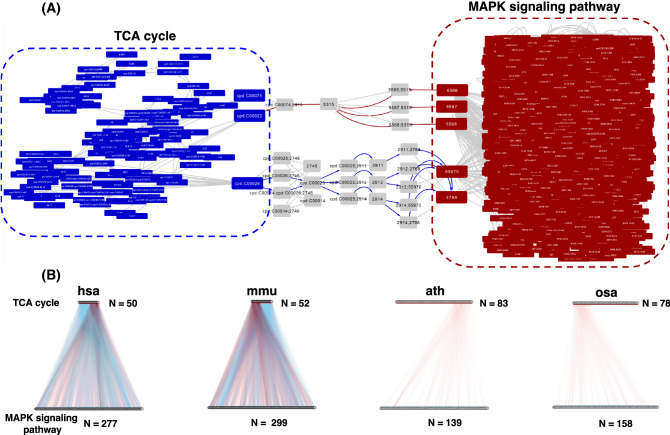
Crosstalk analysis of TCA cycling and MAPK signaling pathways. **A** The two shortest paths in/out of TCA cycling. Blue edges, the paths flowing from TCA cycling pathway to MAPK signaling pathway. Red edges, the paths flowing from MAPK signaling pathway to TCA cycling pathway. Gray edges, the relations between the presented nodes but not in the two paths. **B** The bipartite display of the connections between the nodes of TCA cycling and MAPK signaling pathways. The width of each line was weighted according to the distance between the two nodes (shorter distance was given larger width). The blue and red color of the lines represent the direction of the connections: blue, from TCA cycling pathway to MAPK signaling pathway; red, vice versa

To demonstrate the use of GINs in crosstalk analysis between signaling and metabolic pathways, we interrogated the connections between TCA cycling pathway and MAPK signaling pathway. The members of the two pathways were extracted from the kgml files, respectively. Treating GINs as directed graphs, we calculated the shortest paths between each pair of nodes from the two different pathways, and manually curated the ambiguous connections between extra-cellular molecules and cell-surface receptors extracted from the KEGG database. For the results of human, we built a sub-graph from GIN to illustrate the crosstalk between TCA cycling pathway and MAPK signaling pathway by retaining the shortest path in and out of TCA cycling pathway (Fig. 4A). The paths from TCA cycling to MAPK signaling were through the compound C00026 (2-oxoglutarate), which were converted by gene 2746 (GLUD1) into C00025 (glutamate). Glutamate then bound its receptors (2911, GRM1; 2912, GRM2; and 2914, GRM4) and activate downstream proteins in MAPK signaling pathway. Notably, the binding of glutamate to GRM1/GRM2/GRM4 is usually considered to be extra-cellular. On the other hand, the paths from MAPK signaling to TCA cycling were through three cAMP receptors: 5566 (PRKACA), 5567 (PRKACB), and 5568 (PRKACG), which inhibited 5315(PKM). PKM then catalyzed the generation of C00022 (pyruvate) from C00074 (phosphoenolpyruvate, PEP), which enters TCA cycling.

To have an overview of all the connecting paths between the two pathways, we arranged the nodes (except intermediate nodes) in bipartite layout for the four species of human, mouse, *Arabidopsis*, and rice, and plotted the weighted connections between these nodes (Fig. 4B, Methods). Obviously in human and mouse, there were ample possible paths connecting the nodes of the two pathways, but in *Arabidopsis* and rice, the connections were scarce. This is possibly due to the lack of knowledge on signaling pathways in KEGG’s plant model. For human and mouse, there were a group of nodes of TCA cycling pathways which showed only paths out of TCA cycling (the blue lines) but no incoming connections. We found that these group of nodes were mostly the protein enzymes catalyzing the metabolic reactions, indicating that these protein enzymes might not receive regulation signals from MAPK signaling at the PTM level. However, the possibility that the expressions of these genes were regulated by the transcription factors involved in MAPK signaling could not be excluded, but was out of the scope of this study.

To conclude, we designed a universal data structure for both signaling and metabolic pathways, and converted the KEGG pathway collections of 7077 species into meta-pathways, then constructed a global integrative network merging the signaling and metabolic pathways for each of them. The GINs of the four-representative species showed that the proteins and compound interactions were highly integrated, with less than 11% of the nodes left in pieces of sub-network. Detailed analysis of the subnet pieces revealed distinct features of the GINs between mammals and plants. Crosstalk analysis of TCA cycling and MAPK signaling pathways showed two closest paths connecting the two pathways in human. Our work integrates the signaling and metabolic pathways at data structure level, enables the topological analysis of the integrated pathway networks, and provides references for crosstalk analysis between signaling and metabolic pathways.

## Discussion

The definitions of nodes and edges in signaling pathways and metabolic processes are completely different in current databases, which leads to the chaos when integrating the signaling and metabolic pathways. As a result, the signaling and metabolic pathways were graphically integrated retaining the original definitions of nodes and edges, which was comprehensible for human but hindered topological analysis on the integrated networks. Here, we present a novel structure of “meta-pathway” which is universally suitable for storing the complex relations of signaling pathways, metabolic processes, subunits of protein complexes, and the activation/inhibition of compounds/proteins. These types of biological processes can be easily integrated after converting the pathway collections to meta-pathways. We processed 7077 species pathway collections from KEGG, which provided a ground resource of global integrative networks for further analysis.

One of the most distinct differences observed from the four species’ GINs is the proportion of the compounds and compounds related intermediates. The high ratio of the compounds and compounds related intermediates in plants suggests that plants may rely on complex metabolite processes compared to signaling pathways to survive in the normal and stressed environments, while mammal cells have more complicated signaling system. However, other explanations also exist, one of which is the different resources, efforts and focus put into the researches of mammals and plants.

The GINs of plants are more fragmented than mammals. This might due to the lack of the knowledge in plant pathways compared to mammals. The respective similarities of the fragments in mammals and plants may be due to the fact that mammals and plants have different pathway models in KEGG, and these fragments reflect the scattered knowledge of these models. We believe that with the combination of other pathway databases and the increase of the pathway knowledges, the gaps between the current subnet will be eventually filled and pathways will be assembled into a complete global network.

The GINs integrate signaling and metabolic pathways, however, there are many other types of biological processes in cells to facilitate the coordinated change of cellular state. One important and well-established regulation network is the TF meditated gene regulatory network (GRN), which is associated with all coding gene’s expression. Moreover, TFs are also the “destinations” of the signaling pathways, since the signaling cascades converge on TFs to modulate the gene expression pattern in response to the extra-/intra- cellular signals. Integration of the GRN into the GIN will greatly extend the scope of the network, however, several difficulties must be addressed: the TFs’ effect on gene expressions is variable depending on the tissue, cell type and developmental stages, exemplified by the use of SCENIC to quantify the activity of each regulon in each cell using single-cell RNA-seq data (Aibar et al. 2017). Therefore, for each species, a large number of GRNs are needed to accurately depict the regulatory effect of TFs on the target genes in each cell type. With the development of single-cell omics, this becomes possible for the human model, since single-cell RNA-seq databases of comprehensive cell types in human are now available. However, for other species with less resources and research focus, construction of the GRNs for each cell types are still far from realistic. Another difficulty lies after the integration of GRN in GIN. Since GRN links TFs to all coding genes, there will be a large expansion of the nodes representing new genes in the network after integration. These new genes will be related to various biological processes, which may not be well defined at present. Without sufficient knowledge support, the extended nodes by integrating GRN will still be scattered and unorganized. Nevertheless, integration of GRN in GIN will be of great help to complete the global network of biological processes in cells, and may finally become the framework of a digital model to compute and predict the gene expressions and the changes of cellular states.

Extra-cellular signaling is also an important aspect of cell signaling. Recent tools available for cell–cell interactions, i.e., CellChat (Jin et al. 2021), CellPhoneDB (Efremova et al. 2020), etc., have unveiled the extra-cellular signaling between different cell groups based upon single-cell RNA-seq data. Integration of such network in GIN will extend the network from intra-cellular signaling to extra-cellular signaling, and generate a more comprehensive map of cell signaling.

With further efforts continuously put into the extension of the GIN, it will become more comprehensive and capable of interpreting the multi-omics data. Notably, the introduction of intermediate nodes for multi-omics data will significantly complicated the network, and this expansion will increase the difficulty of interpreting the multi-omics results. However, these difficulties can be partly overcome by avoiding the intermediate nodes of basic machineries such as transcription and translation. For example, Pol II’s role in the transcription of most of the coding genes is universal; therefore, it is not necessary to include Pol II in the intermediate nodes for every gene’s transcription. On the other hand, the introduction of intermediate node has the capacity of displaying complicated relations between TFs, DNA methylations, and histone modifications etc. For example, the binding of a TF with its target gene can be displayed by the forming of an intermediate node of the target’s genomic region with the TF, then this intermediate node can point to the RNA of the gene. Additionally, the DNA methylation of the gene’s promoter can form a node which inhibits the former intermediate node of TF binding. This type of expansion will help the interpretation of multi-omics results at the expense of increased complexity. With the development and maturation of the single-cell multi-omics detection methods, the expanded global integrative network will provide a basic framework for the analysis of the interactions between different data dimensions, i.e., RNA expression, protein expression, metabolites, TF binding, etc., and the interpretation of complicated relations between these dimensions.

## Materials and methods

### Processing of the reactions in KEGG pathways

The kgml files containing KEGG pathway information were downloaded by the function of keggGet from R package “KEGGREST” version 1.30.1. The metabolic reactions were extracted by matching for the entries of “reaction”, and the reaction ID were used to link the corresponding enzymes of the specific species to the substrates and products. The reversible reactions were split into two reactions with inverted directions. Since there are cases in which the recorded enzymes of reactions are actually the subunits of protein complexes, we compiled the protein complex references of each species and assembled the subunits into protein complexes for the reactions. If there are multiple enzymes catalyzing the same reactions, they will be linked to their specific intermediates created using the enzyme and the substrates, respectively, resulting in multiple intermediates with same substrates but different enzymes. Additionally, we observed that in kgml files, usually only the most relevant molecules are recorded. Molecules, especially the commonly used/produced cofactors/products such as ATP/ADP, are not presented in the kgml files except for their biosynthesis pathway. Since inclusion of these molecules may introduce connections between pathways that are not functionally related and disrupt the crosstalk analysis between pathways, we only kept the reactants and products presented in the kgml files instead of searching for all of them by the reaction ID. Finally, all of the four types of relations in Fig. 1A were stored in simple interaction file (SIF or.sif) format, with two columns of the start and the end of the edges, and one column specifying the type of relations.

### Construction of the protein complex references

The information of protein complexes was extracted from the kgml files by matching the for the entries of “group”. The entries of groups specify several entries of the group members, which may include several candidate subunits. For example, protein complex A + (B/C) + D is stored as a group of three members, the protein A, protein B or protein C, and protein D. To avoid chaos, we split this type of protein complex information into complex ABD and ACD. The application of this strategy was carried out using customized tree structure written in perl. Of note, homodimers were ignored in our strategy and stored as one single enzyme unit.

### Processing of the signaling cascades in KEGG pathways

For the signaling cascades, we extracted the relations of protein–protein relations “PPrel” and protein–compound relations “PCrel” from the kgml pathway files. Similar to the processing of reactions, we scanned and assembled the subunits into protein complexes, then stored the four types of relations in SIF format.

### Construction and presentation of the GINs

We merged the meta-pathways of each of the 7077 species and removed the redundant relations which were resulted from the overlapping relations in different pathways. The resulting 7077 GIN files were in SIF format, which is compatible for many network displaying and analysis tools, i.e., cytoscape (Shannon et al. 2003) and visant (Hu et al. 2004), and can be easily manipulated for loading into other tools such as igraph. Of note, we used cytoscape for the presentation of the GINs of the four species in “prefuse forced directed” layout. The processing pipeline was written in Perl and R.

### Analysis of the sub-networks of the four species

Sub-networks with more than 10 nodes were labeled numerically. To analyze the similarities between the sub-networks of different species, we first converted the genes of each sub-network into the corresponding KEGG orthology IDs (the ko number), using the keggGet function from the KEGGREST package and customized R script. The similarities were leveraged by the Jaccard score, defined by the ratio of the number of shared elements divided by the number of elements in the union of two sets:

$$Jaccard\; score=\frac{A\cap B}{A\cup B},$$

where A and B are two sub-networks composed of ko numbers and compound ids. The annotation of the sub-networks was done by using the search function of KEGG mapper tools, since common pathway enrichment tools do not consider the compounds. The naming of the specific sub-networks was done manually, since all of the sub-networks were only small pieces of the pathways.

### Crosstalk analysis of TCA cycling and MAPK signaling pathways

The SIF files of the GINs of the four species were loaded into R, and were treated as edge lists to create igraph objects using “graph_from_edgelist” function from R package “igraph”. Since we observed several ambiguous and misleading information, such as the activation of oxytocin receptor (OXTR) by succinate (generally speaking it should be oxytocin (OXT)) extracted from the kgml file of cAMP signaling pathways, we manually curated the GINs to remove these relations. Furthermore, we also observed that in the graph of glutamatergic synapse (https://www.kegg.jp/pathway/map04724+C00064), the compound labeled “cAMP” activate PKA but actually links to C00064 (L-glutamine), which will result in an additional path linking TCA cycling and MAPK pathways. Since we didn’t find any direct evidence supporting the activation of PKA by L-glutamine, we removed this protein-compound relation in our GINs.

Then we calculated the shortest paths of each pair of nodes between the two pathways. We retained the shortest paths in each of the two directions, and the related nodes for Fig. 4A. For Fig. 4B, the weight of each line was calculated by:

$$W=(\frac{2}{{n}_{\mathrm{shortest}}}{)}^{2},$$

where *W* was the weight, *n*_shortest_ was the number of nodes of the shortest path. The square of the value could suppress the display of the longer paths.

## Supplementary Information

Below is the link to the electronic supplementary material.

Supplementary file1 (PDF 545 KB)

## Data Availability

The data generated during the process of this work (including the 7077 GINs) are available upon request. The codes for this paper are freely available on https://github.com/BIGchix/GINDeclarations
