# Supplementary material for: The global integrative network: integration of signaling and metabolic pathways
Source: aBIOTECH. 2022 Sep 21;3(4):281–91. doi: 10.1007/s42994-022-00078-1 (PMC9755797; doi:10.1007/s42994-022-00078-1)
Supplement: Supplementary file 1 — Supplementary file1 (PDF 545 KB) [file 42994_2022_78_MOESM1_ESM.pdf]

Online resource file 1, for aBIOTECH

# The Global Integrative Network: integration signaling and metabolic pathways

Yu-ying Lin[1], Shen Yan[2], Xiao Chang[1], Xiaoquan Qi[3,4*], Xu Chi[5*].

[1]Department of Dermatology, Xuan Wu Hospital, Beijing, 100053, China.

[2]Agricultural Information Institute, Chinese Academy of Agricultural Science, Beijing, 100081, China

3Key Laboratory of Plant Molecular Physiology, Institute of Botany, Chinese Academy of Sciences, Beijing, 100093, China

[4]The Innovative Academy of Seed Design, Chinese Academy of Sciences, Beijing, 100101, China

[5] CAS Key Laboratory of Genomic and Precision Medicine, Beijing Institute of Genomics, Chinese Academy of Sciences and China National Center for Bioinformation, Beijing, 100101, China

Correspondence: xqi@ibcas.ac.cn (Xiaoquan Qi), chix@big.ac.cn (Xu Chi)

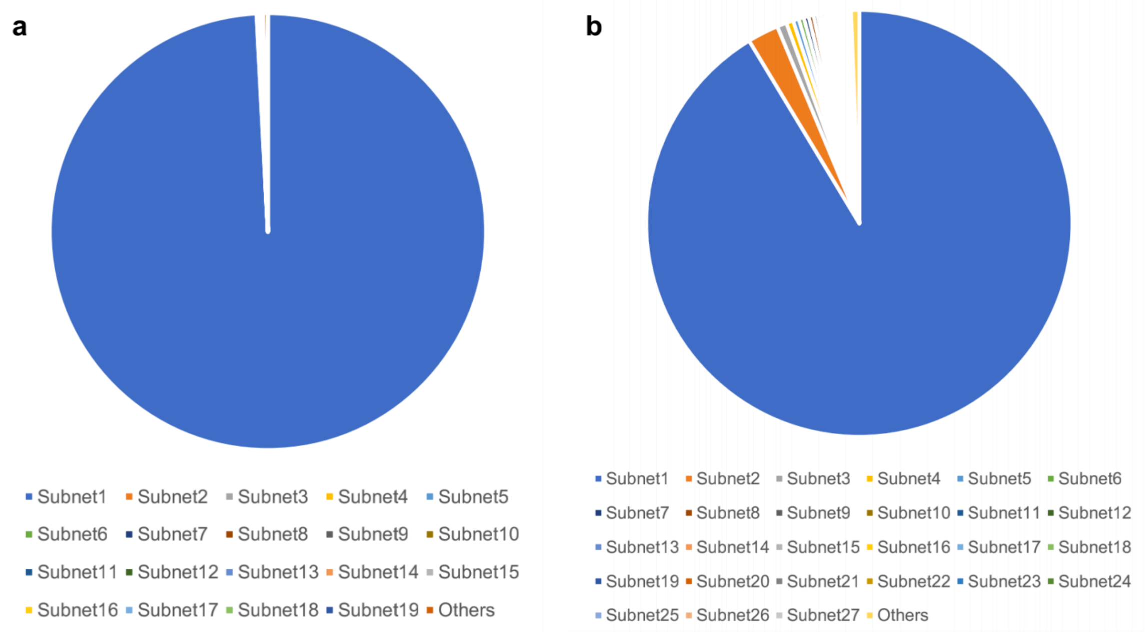


**Fig. S1** The percentage of nodes for each subnet. **a** The percentage of the number of nodes in *Mus musculus*. The percentage of subnet1 is 99.18%. **b** The percentage of the number of nodes in *Oryza sativa*. The percentage of subnet1 is 91.35%, and the percentage of subnet2 is 2.36%

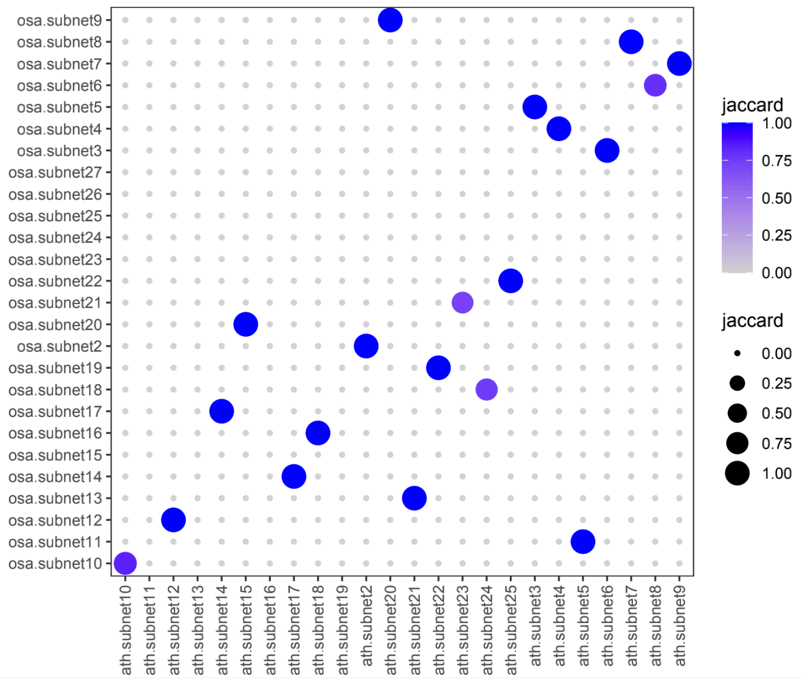


**Fig. S2** The similarities between the subnets of rice and Arabidopsis. Genes were converted into the corresponding ko IDs to enable the calculation of Jaccard score. The compounds were also taken into account during the calculation
